# Intravesical Chemohyperthermia vs. Bacillus Calmette-Guerin Instillation for Intermediate- and High-Risk Non-muscle Invasive Bladder Cancer: A Systematic Review and Meta-Analysis

**DOI:** 10.3389/fsurg.2021.775527

**Published:** 2021-11-23

**Authors:** Hongda Zhao, Vinson Wai-Shun Chan, Daniele Castellani, Erica On-Ting Chan, William Lay Keat Ong, Qiang Peng, Marco Moschini, Wojciech Krajewski, Benjamin Pradere, Chi-Fai Ng, Dmitry Enikeev, Nikhil Vasdev, Gokhan Ekin, Alejandro Sousa, Juan Leon, Felix Guerrero-Ramos, Wei-Shen Tan, John Kelly, Shahrokh F. Shariat, J. Alfred Witjes, Jeremy Yuen-Chun Teoh

**Affiliations:** ^1^S.H. Ho Urology Centre, Department of Surgery, The Chinese University of Hong Kong, Shatin, Hong Kong SAR, China; ^2^School of Medicine, Faculty of Medicine and Health, University of Leeds, Leeds, United Kingdom; ^3^Unit of Urology, Azienda Ospedaliero-Universitaria Ospedali Riuniti di Ancona, Polytechnic University of the Marche Region, Ancona, Italy; ^4^Department of Urology, Penang General Hospital, George Town, Malaysia; ^5^Klinik für Urologie, Luzerner Kantonsspital, Lucerne, Switzerland; ^6^Department of Urology and Oncologic Urology, Wrocław Medical University, Wrocław, Poland; ^7^Department of Urology, University Hospital of Tours, Tours, France; ^8^Institute for Urology and Reproductive Health, Sechenov University, Moscow, Russia; ^9^Department of Urology, Hertfordshire and Bedfordshire Urological Cancer Centre, Lister Hospital Stevenage, School of Medicine and Life Sciences, University of Hertfordshire, Hatfield, United Kingdom; ^10^Department of Urology, Urla State Hospital, Izmir, Turkey; ^11^Department of Urology, Comarcal Hospital, Monforte, Spain; ^12^Department of Urology, University Hospital 12 de Octubre, Madrid, Spain; ^13^Division of Surgery and Interventional Sciences, University College London, London, United Kingdom; ^14^Department of Urology, University College London Hospital, London, United Kingdom; ^15^Department of Urology, Royal Free Hospital, London, United Kingdom; ^16^Department of Urology, Medical University of Vienna, Vienna, Austria; ^17^Department of Urology, Weill Cornell Medical College, New York, NY, United States; ^18^Department of Urology, University of Texas Southwestern, Dallas, TX, United States; ^19^Department of Urology, Charles University, Prague, Czechia; ^20^Division of Urology, Department of Special Surgery, Jordan University Hospital, The University of Jordan, Amman, Jordan; ^21^Department of Urology, Radboud University Medical Centre, Nijmegen, Netherlands

**Keywords:** bladder cancer, TURBT (trans-urethral resection of bladder tumour), BCG–Bacillus Calmette-Guérin vaccine, chemohyperthermia, meta-analysis

## Abstract

**Background:** The efficacy of intravesical chemotherapy maintenance for patients with non-muscle invasive bladder cancer (NMIBC) is inferior compared to intravesical bacillus Calmette–Guerin (BCG). How intravesical chemohyperthermia (CHT) compares with BCG is under investigation.

**Objective:** To compare the oncological outcomes and safety profile between intravesical CHT and BCG treatment for intermediate- and high-risk NMIBC.

**Methods:** We performed a systematic review and meta-analysis of clinical studies comparing CHT with BCG for intermediate- and high-risk NMIBC patients. A comprehensive literature search on OVID MEDLINE, EMBASE, and Cochrane Library was conducted. Risk of bias was assessed by the Cochrane RoB tool and ROBINS-I. Certainty of evidence was rated using the Grading of Recommendations Assessment, Development and Evaluation (GRADE) methodology.

**Results:** A total of 2,375 articles were identified and five studies were finally included. Among them, four randomised trials comprising 327 patients (CHT group: 156 patients; BCG group: 171 patients) were included in the meta-analysis. There were no significant differences in the 24–36 months recurrence rates (CHT: 29.5%, BCG: 37.4%; RR: 0.83, 95% CI 0.61–1.13; moderate certainty of evidence) and the 24–36 months progression rates (CHT: 4.4%, BCG: 7.6%, RR = 0.62, 95% CI 0.26–1.49; low certainty of evidence). There were also no significant differences in grade 1–2 adverse events (CHT group: 59.9%, BCG group 54.5%; RR = 1.10, 95% CI 0.93–1.30; moderate certainty of evidence) and grade 3 or above adverse events (CHT group: 23.2%, BCG group 22.5%; RR = 0.99, 95% CI 0.69–1.43; low certainty of evidence).

**Conclusions:** Intravesical CHT had equivalent oncological outcomes and similar safety profile when compared to BCG maintenance therapy for patients with intermediate- and high-risk NMIBC. CHT is a possible alternative treatment in the times of BCG shortage.

## Introduction

Bladder cancer is the 11th most common cancer worldwide, and more than 75% of the patients present with non-muscle invasive bladder cancer (NMIBC) (1, 2). Transurethral resection of bladder tumour (TURBT) is a potentially curative surgery, yet the oncological control of NMIBC is unsatisfactory with a one-year recurrence rate of up to 31%, and a five-year recurrence rate of up to 78% (3, 4).

NMIBC is classified into low-risk, intermediate-risk, and high-risk disease based on its clinical and pathological factors (5, 6). For intermediate- and high-risk NMIBC, intravesical bacillus Calmette–Guerin (BCG) therapy has been shown to be effective in reducing disease recurrence and progression (7). On the other hand, intravesical BCG therapy is associated with local and systemic toxicities, and it may not be well-tolerated throughout the whole treatment course (8, 9). Moreover, BCG shortage is a significant global problem (10). There is an urgent need to seek for an alternative treatment that is at least equally effective, and with better tolerability and secured availability for patients with intermediate- and high-risk NMIBC (11).

Intravesical maintenance chemotherapy has long been investigated in patients with NMIBC. Although it was associated with a lower rate of adverse events, its treatment efficacy has been proven to be inferior to intravesical maintenance BCG therapy (12, 13). In recent years, there has been increasing use of adjuvant intravesical chemohyperthermia in NMIBC patients. By increasing the temperature of chemotherapy (Combat System) or the bladder wall (Synergo system) to 42–43°C degrees, may enhance its drug absorption and cytotoxic effects (14). Although intravesical CHT is a promising treatment, its distinction of treatment outcome comparing with BCG is not well-known. In this systematic review, we aim to investigate the treatment efficacy and adverse events of intravesical CHT vs. BCG in patients with intermediate- and high-risk NMIBC.

## Methods and Materials

A systematic review and meta-analysis was conducted according to the Preferred Reporting Items for Systematic Reviews and Meta-Analyses (PRISMA) statement (15). The study protocol was registered on the international prospective register of systematic reviews (PROSPERO) (Registration number: CRD42020223277).

### Literature Search

We conducted a comprehensive literature search on OVID MEDLINE, EMBASE, and Cochrane Central Controlled Register of Trials (CENTRAL), using Medical Subject Headings (MeSH) terms and keywords related to “Bladder cancer,” “Bacillus Calmette-Guérin,” and “Chemohyperthermia.” The search was performed from database inception up to the 1st of September 2020. All full-text publications, conference abstracts and proceedings in English language were included. Reference lists of the included studies were sought for additional articles. The search strategy is presented in Supplementary Material.

### Selection Criteria

Randomised controlled trials (RCTs) and observational studies comparing the use of CHT and BCG instillation in intermediate- or high-risk NMIBC patients post-TURBT were included. Only human studies were included and there was no limit to the type of CHT device used. Editorials, commentaries, reviews, case reports, case series and single arm studies were excluded. Studies comparing the use of CHT and normothermic chemotherapy were also excluded.

### Screening and Data Extraction

All identified articles were initially screened by two independent reviewers by title and abstract. Conflicts were resolved by a third senior author. Full texts of potentially eligible studies were then retrieved for further screening in the same manner. Finally, a standardised and piloted data extraction form was devised to capture data such as baseline characteristics of studies, details of intervention and control, along with outcomes of interest. The corresponding authors of each study with missing data were contacted in order to retrieve any missing data.

### Data Synthesis and Statistical Analysis

The primary outcomes of our study included recurrence and progression rates at 24–36 months. Secondary outcomes included recurrence-free survival (RFS), progression-free survival (PFS), grade 1–2 and grade 3 or above adverse events (AEs) according to the National Cancer Institute Common Terminology Criteria (16). Meta-analyses were only performed when there were two or more RCTs reporting the same outcome under the same definition. Rates of recurrence, progression and AEs were analysed as dichotomous events using the Mantel-Haenszel method, and were reported as risk ratios (RR), 95% CIs and *p*-values. For RFS and PFS, hazard ratios (HR) and 95% Confidence Interval (95% CI) derived by the Cox Proportional hazards model were pooled using the inverse variance method, and were reported as HRs, 95% CIs and *p*-value. In studies where Hazard Ratios were not reported, HRs are estimated using validated methods outlined by Tierney et al. (17) as recommended by the Cochrane Collaboration (18). The random effects (RE) model was used to take into account substantial heterogeneity where identified, otherwise, the fixed effects (FE) model was used. Heterogeneity was assessed using the Cochran's *I*^2^, and substantial heterogeneity was defined as an *I*^2^ value >50% or a Chi^2^ *p*-value <0.10. Owing to the potential source of heterogeneity originating from the types of CHT used, subgroup differences were tested between the major types of CHT used, and was defined as a Chi^2^ *p*-value <0.10. Planned sensitivity analyses were also performed on patients without BCG failure and without carcinoma *in situ* (CIS) diseases. All data-analyses were performed using Review Manager v.5.4. Results from non-randomised studies were summarised narratively. Risk of bias of RCTs was assessed using the Risk of Bias 2.0 tool as recommended by the Cochrane Collaboration (19, 20). Risk of bias in non-randomised studies were assessed using the non-randomised studies–of interventions (ROBINS-I) tool (21). Summary of findings for all outcomes, along with the certainty of evidence which was rated according to the Grading of Recommendations Assessment, Development and Evaluation (GRADE) approach (22) were tabulated using the GRADEpro tool (23).

## Results

### Literature Search Results

**Figure 1:**
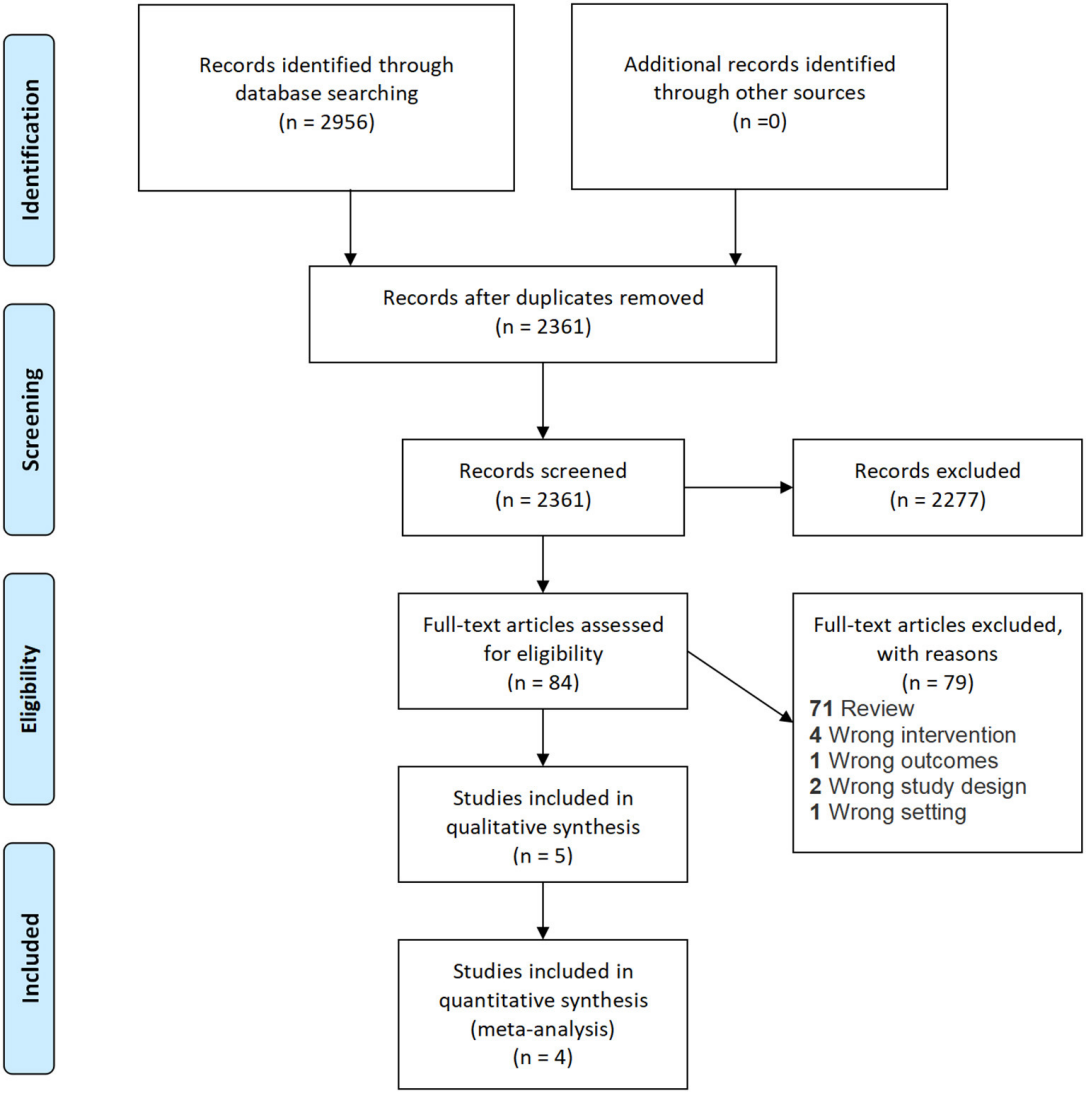
PRISMA flow diagram.

The PRISMA flow diagram is shown in Figure 1. A total of 2,956 records were identified upon the literature search. No additional records were identified during screening of reference lists of included articles. 2,361 abstracts remained after removal of duplicates. A total of 2,277 articles were excluded upon initial screening, and 79 studies were further excluded upon full-text screening. Finally, four RCTs (24–27) were included for meta-analysis, and one observational retrospective study (28) was retrieved and included for qualitative synthesis. Two studies included both intermediate- and high-risk NMIBC patients (24, 25), and the other three studies included high-risk NMIBC patients only (26–28). All studies were non-inferiority trials and did not specifically focus on primary or recurrent cases. All five studies had similar follow-up durations of 24–36 months. The study information of the five studies is shown in Table 1. The risk of bias assessment and the GRADE summary of finding profiles are included in Supplementary Material.

**Table 1:** Characteristics of the included studies.

**Study (year)**	**Country of study**	**Study design**	**Number of centres**	**Recruitment period**	**Duration of follow up (months)**	**Inclusion and exclusion criteria**	**Number of patients (Intervention/ control)**	**Age (intervention/ control)**	**Sex (M/F)**	**Device used**	**Regime for CHT**	**Regime for BCG**
Sousa 2020	Spain	RCT	2	Between March 2015 and June 2019	Mean: 38	1. Histological confirmed previous UCC 2. NMIBC following recurrence of G1-3 pTa or G1-2 pT1 3. Tumour number ≤ 6 number of tumours 4. Aged ≥18 years 5. No solid tumour, muscle infiltrating aspect or CIS suspicious, positive cytology and recurrence of previous T1G3 or CIS tumours in the last 12 months	16/17	Mean ± SD:71 ± 3.2/69 ± 2.7	27/6	Combat BRS system	Weekly for 8 weeks, 80 mg MMC	NA
Guerrero- Ramos 2020	Spain	RCT	1	NR	Median: 24.8	1. NMIBC 2. No CIS 3. No intolerance or contraindication for receiving BCG or MMC	24/24	Entire group mean: 73	42/6	Combat BRS system	Weekly for 6 weeks; follow by monthly for 6 months, 40 mg MMC	Weekly for 6 weeks and maintenance according to SWOG protocol.
Ekin 2015	Turkey	Retrospective cohort study	2	Between January 2004 and January 2014	Median(IQR): 33(24–39)	1. High-risk of NMIBC treated with intravesical C-HT or BCG instillation 2. Performed second-TUR 3. Not treated with reduced dose of BCG, 4. No bladder diverticulum >1 cm 5. No histopathology non-urothelial carcinoma 6. No concomitant urothealial carcinoma in the urethra or upper urinary tract 7. No low bladder capacity (<150 mL) 8. No high post-voided residual urine (>100 mL)	39/39	Mean ± SD (range):68.05 ± 9.29 (47–84)/ 68.02 ± 8.42 (48–82)	73/5	Elmedical technologies BWT	Weekly for 6 weeks; Also 3 weekly instillations at month 3 and month 6. 40 mg MMC	Weekly for 6 weeks. The choice of maintenance was determined by the physician and/or patient.
Arends 2016	Israel Italy, the Netherlands Austria, France, Belgium	RCT	11	Between 18 July 2002 and 25 December 2011	Median(range): 25.6 (0.0–34.0)	1. pT1 or grade3 UCC and/or CIS or multifocal (six or more) pTa lesions and/or multiple (three or more) recurrences of pTa lesions in the last 24 months 2. WHO performance status ≤ 2, 3. Life expectancy >24 months 4. No histopathology non-urothelial carcinoma (basal cell carcinoma excluded) 5. No UCC involving the urethra or upper urinary tract 6. No previous history of UCC stage T2 or higher 7. No intravesical MMC treatments during the previous 12 months 8. No previous BCG therapy <48 mo 9. No previous pelvic radiotherapy, systemic chemotherapy or partial cystectomy 10. No bladder diverticulum >1 cm, residual urine >100 ml, bladder volume <150 ml, urinary incontinence, urethral stricture impeding 20F catheterization 11. No persistent haematuria 12. No active intractable or uncontrollable UTI, active tuberculosis or BCG infection	89/95	Mean ± SD:65.2 ± 10.67/ 67.4 ± 10.08	154/30	Synergo system	Weekly for 6 weeks, followed by 6 maintenance sessions at 6-wk intervals during the rest of year 1. Two 30-min treatments with 20 mg MMC	Six weekly induction sessions and three weekly repeated maintenance sessions at months 3, 6, and 12
						13. No previous BCG life-threatening sepsis, MMC or BCG allergy, impaired immune response, positive HIV serology, receipt of systemic steroids or immunosuppressives 14. No haematological disorders, leukocytes <3500, platelets <100 000, kidney or liver function disorders (>1.5 times upper normal limit), and pregnant/lactating.						
Tan 2019	UK	RCT	14	Between May 2010 and July 2013	Median:36	1. Recurrence of intermediate- or high-risk NMIBC following induction/maintenance BCG 2. Having complete TUR of papillary lesions 3. pT1 disease underwent re-resection to confirm the absence MIBC 4. Age ≥18 years 5. WHO performance status ≤ 4 6. Unfit or unwilling to have radical cystectomy 7. Imaging showed no upper tract disease ≤ 12 mo. 8. Haematological and biochemical blood tests were within normal limits 9. No non-urothelial carcinoma 10. No low-grade NMIBC recurrence	48/56	Median (IQR)77 (72–82)/76 (67–81)	78/26	Synergo system	Weekly for 6 weeks Patients who were disease -free 3 mo after treatment commencement would proceed to maintenance RITE (one instillation of RITE every 6 wk for 1st yr and one instillation every 8 wk for 2nd yr). Two 30-min cycles, each with 20 mg MMC	Six consecutive weekly instillations followed by maintenance therapy (three consecutive weekly instillations at 3, 6, 12, 18, and 24 mo)
						11. No treatment with intravesical chemotherapy ≤ 6 mo (single post-TUR instillation allowed) 12. No prostatic urethra or upper tract disease 13. No MMC allergy 14. No active/intractable urinary tract infection 15. No urethral stricture, small bladder capacity (<250 ml), significant urinary incontinence, or history of pelvic radiotherapy.						

*WHO, World Health Organisation; NR, not reported; UCC, urothelial cell carcinoma; NMIBC, non-muscle invasive bladder cancer; CIS, Carcinoma in situ; MMC, mitomycin C; BCG, bacillus Calmette-Guerin; UTI, urinary tract infection; TUR, transurethral resection*.

### Study Outcomes

#### Recurrence Rate at 24–36 Months

**Figure 2:**
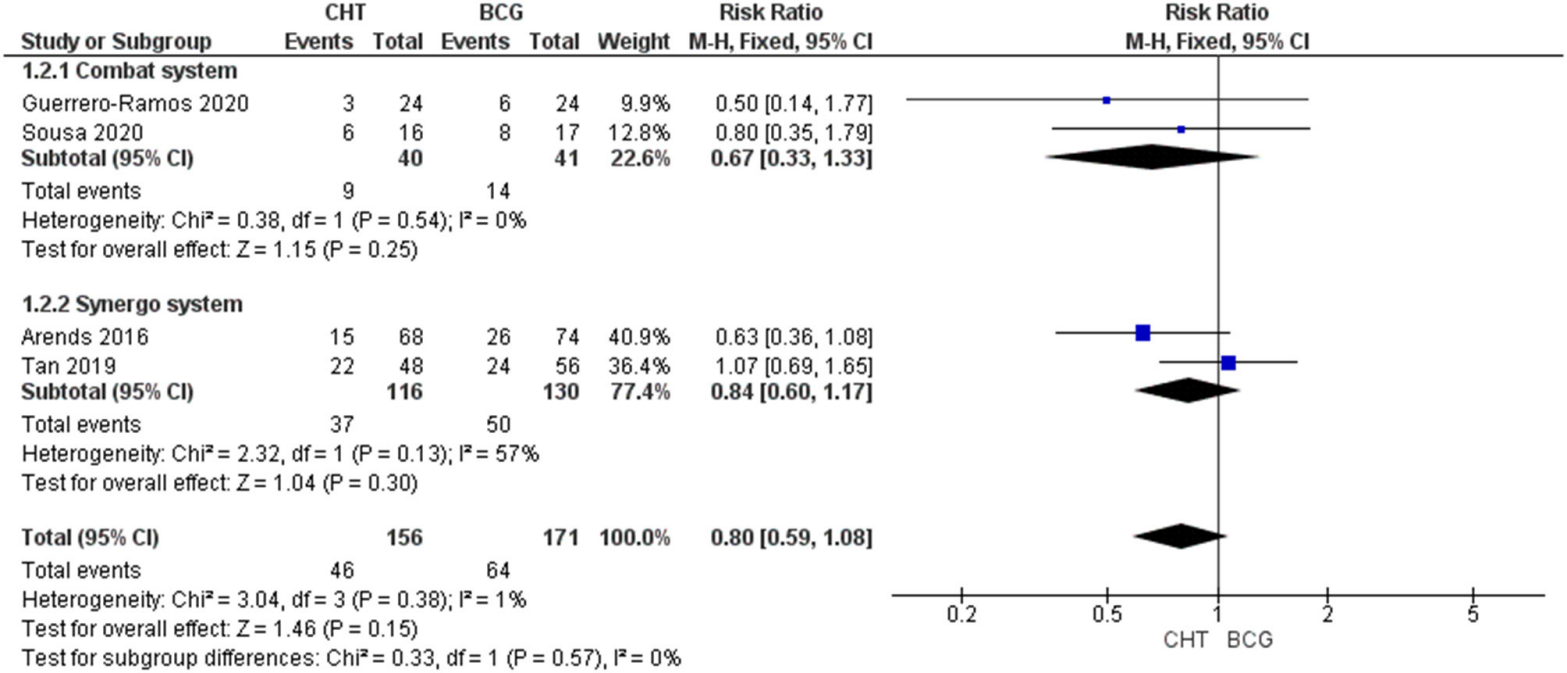
24–36 months recurrence rate stratified by the type of chemohyperthermia device.

Four RCTs with 327 patients were included (24–27). There was no significant difference between the two groups (RR_FE_ 0.80, 95% CI 0.59–1.08; moderate certainty of evidence) (Figure 2). No significant heterogeneity (*I*^2^ = 1%, *p* = 0.38) was detected. Upon subgroup analysis, no differences were found between the use of conductive hyperthermia and radiofrequency-induced thermochemotherapeutic effect (RITE). Sensitivity analysis after excluding BCG failure patients from the HYMN study shows CHT has a significantly lower recurrence rate when compared to BCG group (RR_FE_: 0.64, 95% CI 0.42–0.98, *p* = 0.04) (Supplementary Material). Of note, in the RCT by Sousa et al. (26), the conductive CHT group had significantly lower rate of recurrence when compared to the BCG group (20.5% vs. 38.2%, *p* <0.02). However, a retrospective matched cohort study by Ekin et al. (28) found a significantly higher recurrence rate in patients receiving conductive CHT compared to those who received BCG (35.9% vs. 20.5%, *p* <0.05).

#### Progression Rate at 24–36 Months

**Figure 3:**
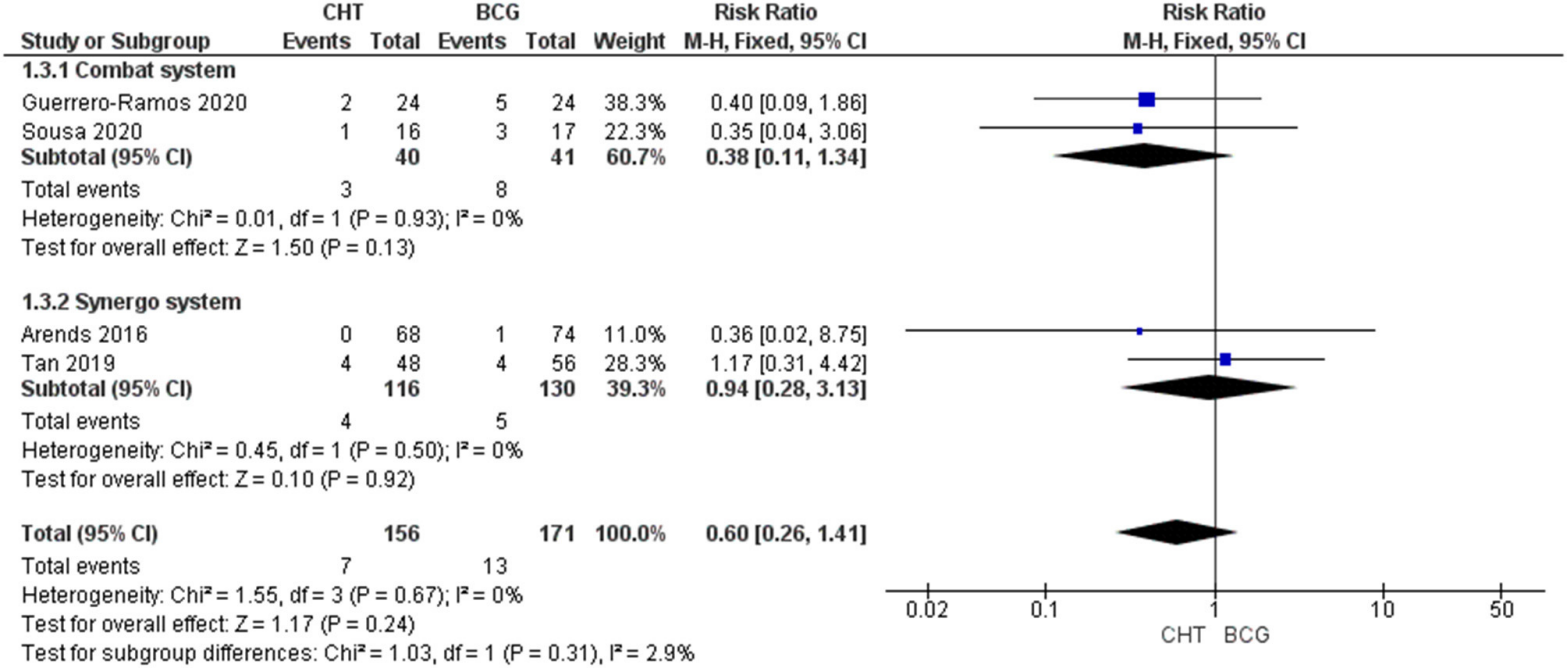
24–36 months progression rate stratified by the type of chemohyperthermia device.

Four RCTs with 327 patients were included (24–27). There was no significant difference between the two groups (RR_FE_ 0.60, 95% CI 0.26–1.41, *p* = 0.24; low certainty of evidence) (Figure 3). No significant heterogeneity was detected (*I*^2^ = 0%, *p* = 0.67). No significant difference between conductive hyperthermia and RITE was found upon subgroup analysis. When excluding patients with BCG failure, progression rate was also found to be similar in CHT patients when compared to BCG patients (RR_FE_ 0.38, 95% CI 0.12–1.22, *p* = 0.10). Of note, in the RCT performed by Sousa et al. (26), T1 progression and T2 progression were significantly reduced in the conductive system CHT group when compared to the BCG group (*p* <0.05 and *p* <0.01 respectively). However, in a retrospective matched cohort study performed by Ekin et al. (28) the use of conductive CHT was associated with significantly higher progression rate when compared to BCG (15.4% vs. 7.7%, *p* <0.05).

#### Recurrence-Free Survival

Three RCTs were included in the meta-analysis (24, 25, 27). In terms of RFS, no significant difference was noted between the CHT group and the BCG group (HR_RE_ 0.81, 95% CI 0.42–1.56, *p* = 0.53; very low certainty of evidence) (Supplementary Material). However, there was significant heterogeneity amongst the included studies (*I*^2^ = 68%, *p* = 0.04). Our subgroup analysis suggested that this heterogeneity did not originate from the type of CHT systems used; no differences were found between the conductive CHT group and the SRITE group. When performing a sensitivity analysis to exclude patients with BCG failure (i.e., patients from the HYMN trial), RFS is found to be significantly better in CHT patients than BCG patients (HR_RE_ 0.57, 95% CI 0.33–0.98) with no significant heterogeneity (*I*^2^ = 0%, *p* = 0.91) (Supplementary Material), suggesting the potential source of heterogeneity to originate from BCG failure patients. Of note, in a retrospective study by Ekin et al. (28), it was found that the use of conductive CHT was associated with significantly worsened RFS when compared to BCG instillation (HR 4.18, 95% CI 1.37–12.71, *p* = 0.012). However, when performing sensitivity analysis by excluding the HYMN study, where only patients with BCG failures were considered, the remaining two studies show that CHT group has a better RFS when compared to the BCG group (HR_FE_ 0.52, 95% CI 0.29–0.93; Supplementary Figure 1). However, when excluding patients with CIS disease, the RFS is both groups remained similar (HR_FE_ 0.72, 95% CI 0.48–1.09) (Supplementary Material).

#### Progression-Free Survival

Two RCTs were included in the meta-analysis (24, 27). In terms of PFS, there was no significant difference between the CHT group and the BCG group (HR_RE_ 0.92, 95% CI 0.25–3.40; very low certainty of evidence) (Supplementary Material). However, there was significant heterogeneity amongst the included studies (*I*^2^ = 73%, *p* = 0.06). The heterogeneity might originate from the different types of CHT device being used as evident by the test for subgroup differences (*p* = 0.06), but this should be interpreted with caution due to the limited number of studies being included. Furthermore, the study by Tan et al. also included BCG failure and CIS patients, which may have lead to a significantly lower PFS rate. In the retrospective study by Ekin et al. (28), no significant difference was found between the CHT group and the BCG group (HR 1.72, 95% CI 0.28–10.36, *p* = 0.550).

#### Adverse Events

**Figure 4:**
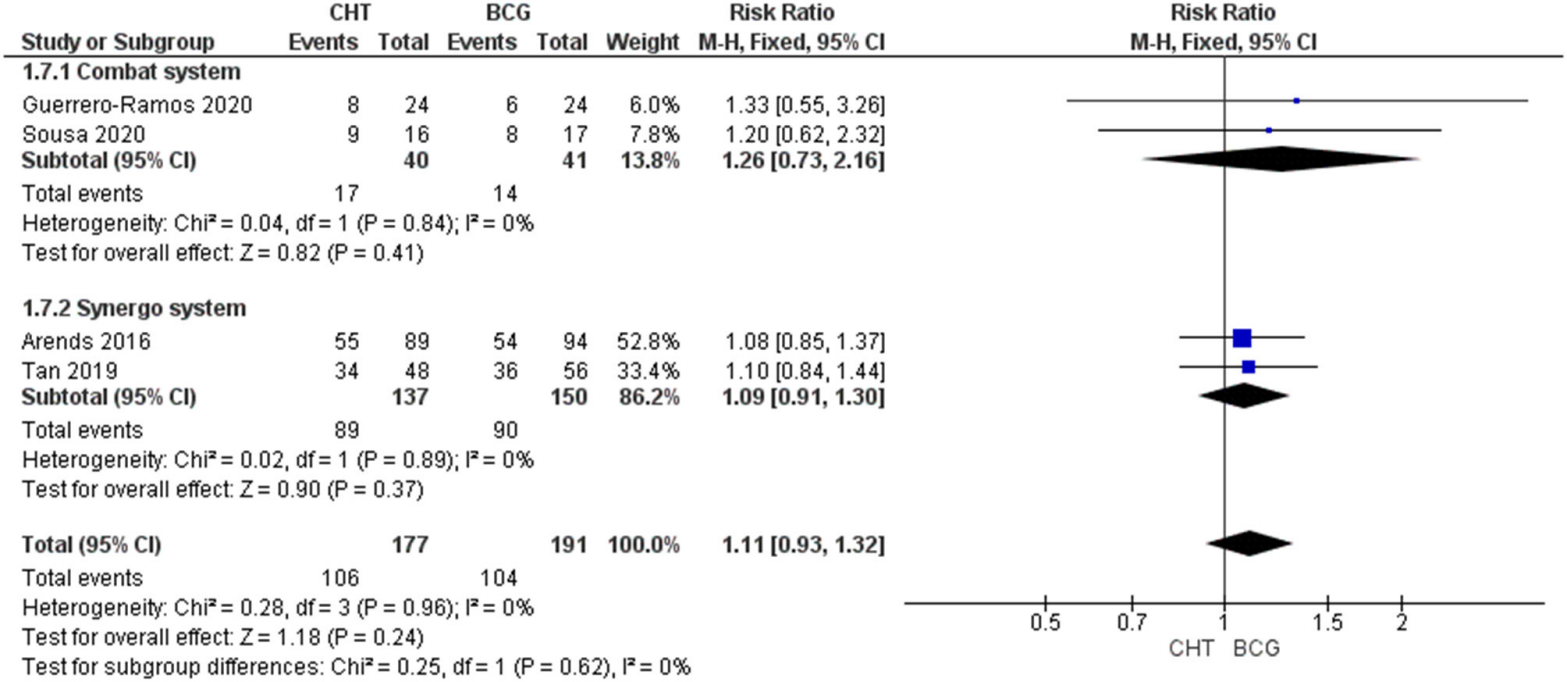
Grade 1–2 adverse events stratified by the type of chemohyperthermia device.

**Figure 5:**
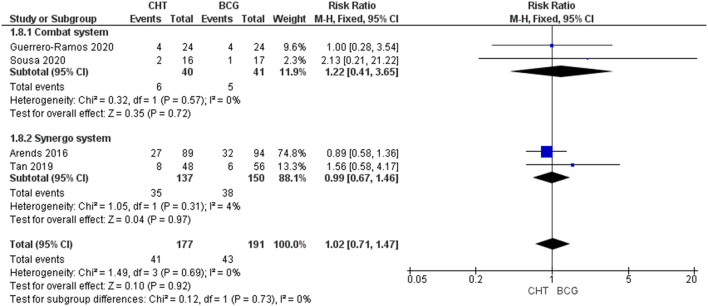
Grade 3 or above adverse events stratified by the type of chemohyperthermia device.

Four RCTs with 368 patients were included (24–27). For Grade 1-2 AEs, there was no significant difference between the CHT group and the BCG group (RR_FE_ 1.11, 95% CI 0.93–1.32, *p* = 0.26; moderate certainty of evidence), and no significant heterogeneity was detected (*I*^2^ = 0%, *p* = 0.96) (Figure 4). For grade 3 of above AEs, there was also no significant difference between the CHT group and the BCG group (RR 1.02_FE_, 95% CI 0.711.47, *p* = 0.92; low certainty of evidence), and no significant heterogeneity was detected (*I*^2^ = 0%, *p* = 0.69) (Figure 5).

## Discussion

Intravesical BCG therapy is a standard treatment for patients with intermediate- and high-risk NMIBC following TURBT (5). However, it is not without limitations. First, more than half of the patients might develop local and systemic toxicities, such as bacterial/chemical cystitis, frequency, haematuria, allergic reactions and BCG sepsis (8, 9, 29). While a minimum duration of 1-year treatment course is recommended, about half of the patients would withdraw from treatment prior to completion of BCG therapy (30, 31). Second, the supply of BCG has been unsteady in the past decade. Globally, there were only a few manufacturers of BCG, and the production of BCG is generally limited by the slow growth of mycobacteria (10). Therefore, it is imperative for researchers to look for alternative treatments for patients with intermediate- and high-risk NMIBC.

Intravesical chemotherapy has been proven to be less effective than BCG (12, 13). However, the development of device-assisted technology could optimise the efficacy of chemotherapy and potentially maintaining its safety and tolerability. In particular, CHT has gained significant traction within the urological community leading to a steadily increasing use in the past decade. The cytotoxicity of chemotherapy can be accentuated when its temperature reaches 42 to 43 degrees (32). Several mechanisms of action of hyperthermia has been postulated to synergistically enhance the efficacy of intravesical chemotherapy. First, hyperthermia alone could cause the denaturation of cytoplasmic structures and enzymatic proteins, thus inducing cell death by apoptosis and necrosis (32–34). Second, temperature elevation could enhance the permeability of cell membrane and improve drug absorption (35, 36). Third, heat shock proteins could be released upon hyperthermia, thus stimulating an adaptive T cell response to induce both innate and adaptive immune system. Tumour chemosensitization may also be achieved via the heat shock proteins-mediated pathways (37, 38).

Delivery of hyperthermia can be achieved by two main methods, namely conductive hyperthermic chemotherapy (Combat system) and RITE (Synergo). For conductive hyperthermic chemotherapy, the chemotherapy solution was heated externally and recirculated to the bladder at a constant temperature. For RITE, microwave radiation was delivered to the bladderwall at a frequency of 915 MHz. Without the need of conductive delivery of energy, it has a potential benefit to penetrate low-conductive tissues (39).

In our study, we compared between intravesical CHT and BCG in patients with intermediate- and high-risk NMIBC. Our results showed that CHT could achieve an equivalent oncological outcome as BCG therapy in terms of recurrence and progression rates at 24–36 months. Our sensitivity analysis would suggest that efficacy was generally consistent across the two different types of CHT technologies, the Combat/ Unithermia system and the Synergo system. One study population was, however, too small to allow statistically powered comparison between the CHT devices. Intravesical CHT is a reasonable treatment option for intermediate- and high-risk NMIBC given its similar efficacy to BCG. Although the use of CHT was associated with additional costs, a more steady supply can be assumed without the worry of BCG shortage. On the other hand, our meta-analysis showed that the rates of grade 1–2, and grade 3 or above AEs were similar between intravesical CHT and BCG. In other words, based on the current evidence, we cannot assume that CHT is safer or more tolerable than BCG therapy. A realistic expectation should be given when we counsel patients on the usage of CHT.

In many parts of the world, intravesical maintenance chemotherapy is the mainstay of treatment for intermediate-risk, and even high-risk NMIBC (40). A recent meta-analysis showed that intravesical CHT was associated with a lower recurrence rate when compared to normothermic chemotherapy. The HIVEC I and HIVEC II studies are both multicentre RCTs comparing between CHT and normothermic chemotherapy in patients with intermediate-risk NMIBC. Initial results on safety and tolerability were comparable between the two groups (41); the final oncological outcomes are eagerly awaited.

To our knowledge, this is the first meta-analysis comparing between CHT and BCG in patients with NMIBC. It is based on a comprehensive literature search including conference abstracts and proceedings, therefore publication bias is minimised. Only data from RCTs were meta-analysed, and the certainty of evidence was determined using the GRADE methodology. On the other hand, there are several limitations in our study. First, only four RCTs were included and the sample size is still relatively limited. More RCTs comparing intravesical CHT to BCG are warranted. Second, some of the included RCTs are still on-going, so the collected data may be premature and may not be reflective of the final results. Third, significant heterogeneity does exist in some of our analysis. This may be due to the differences in the underlying patient cohort characteristics; the results should be therefore interpreted with caution. Further sources of heterogeneity may have been from the definition of high-risk bladder cancer, contributed by the recent change in guidelines as well as potentially different treatment regimens between studies. Finally, while carefully considered using sensitivity analyses, design studies incorporating CIS or papillary disease patients or BCG failure patients may be additional sources of heterogeneity. Nevertheless, our study did shed light on the utility of CHT in patients with intermediate- and high-risk NMIBC. Compared to BCG therapy, intravesical CHT could be an equally effective and tolerable treatment option. Although the utility of CHT implies additional cost, a more “comfortable” treatment regime for patients with a shorter overall treatment time may be preferred. Utility of CHT may also provide a solution to the problem of BCG shortage worldwide. The results have important implications in our clinical practise until higher level of evidence arises.

## Conclusion

Our meta-analysis showed that intravesical CHT had equivalent oncological outcomes and similar safety profile when compared to BCG therapy for patients with intermediate- and high-risk NMIBC. In well-selected patients, i.e., those without BCG failure, CHT is even more superior than BCG maintenance in terms of recurrence rate. Intravesical CHT is a possible alternative treatment in the times of BCG shortage. More RCTs comparing intravesical CHT to BCG are warranted to develop a clearer image of the value-based utility of CHT in this patient population.

## Data Availability Statement

The original contributions presented in the study are included in the article/Supplementary Material, further inquiries can be directed to the corresponding author.

## Author Contributions

HZ and VC: data collection, interpretation of data, and manuscript writing. DC, EC, and C-FN: interpretation of data. WO and QP: data collection. MM, WK, BP, DE, NV, GE, AS, JL, FG-R, W-ST, JK, SS, and JW: raw data providing. JT: data collection, interpretation of data, manuscript writing, and supervising. All authors contributed to the article and approved the submitted version.

## Conflict of Interest

JK and W-ST are investigators of the HYMN trial where SYNERGO provided the system and catheters, SYNERGO was not involved in the conception, design and execution of the trial. The HYMN trials was funded by the Cancer Research UK. JK and W-ST are consultants to Combat Medical. FG-R is an advisor and speaker for Combat Medical. The remaining authors declare that the research was conducted in the absence of any commercial or financial relationships that could be construed as a potential conflict of interest.

## Publisher's Note

All claims expressed in this article are solely those of the authors and do not necessarily represent those of their affiliated organizations, or those of the publisher, the editors and the reviewers. Any product that may be evaluated in this article, or claim that may be made by its manufacturer, is not guaranteed or endorsed by the publisher.
