# Supplementary material for: Intravesical Chemohyperthermia vs. Bacillus Calmette-Guerin Instillation for Intermediate- and High-Risk Non-muscle Invasive Bladder Cancer: A Systematic Review and Meta-Analysis
Source: Front Surg. 2021 Nov 23;8:775527. doi: 10.3389/fsurg.2021.775527 (PMC8649716; doi:10.3389/fsurg.2021.775527)
Supplement: Supplementary file 1 [file Data_Sheet_1.DOCX]

**Supplementary material**

| **Table of Contents** | Page |
| --- | --- |
| 1. Search strategy | 2 |
| 2. Risk of bias assessment for randomised controlled trials | 3 |
| 3. Risk of bias assessment for non-randomised study | 4 |
| 4. Recurrence-free survival stratified by the type of chemohyperthermia device | 6 |
| 5. Progression-free survival stratified by the type of chemohyperthermia device | 7 |
| 6. Recurrence rate in patients without BCG failure | 8 |
| 7. Progression rate in patients without BCG failure | 9 |
| 8. Recurrence-free survival in patients without BCG failure | 10 |
| 9. Recurrence-free survival in patients without CIS disease | 11 |
| 10. Grade summary of the included randomised controlled trials | 12 |
| 11. Grade summary of the included non-randomised study | 15 |

1. Search strategy

Database: EBM Reviews - Cochrane Database of Systematic Reviews <2005 to August 26, 2020>, EBM Reviews - ACP Journal Club <1991 to August 2020>, EBM Reviews - Database of Abstracts of Reviews of Effects <1st Quarter 2016>, EBM Reviews - Cochrane Clinical Answers <August 2020>, EBM Reviews - Cochrane Central Register of Controlled Trials <July 2020>, EBM Reviews - Cochrane Methodology Register <3rd Quarter 2012>, EBM Reviews - Health Technology Assessment <4th Quarter 2016>, EBM Reviews - NHS Economic Evaluation Database <1st Quarter 2016>, Embase Classic+Embase <1947 to 2020 September 01> , Ovid MEDLINE(R) and Epub Ahead of Print, In-Process & Other Non-Indexed Citations, Daily and Versions(R) <1946 to September 01, 2020>

--------------------------------------------------------------------------------

1 exp Urinary Bladder Neoplasms/dt or exp bladder cancer/dt (22019)

2 ((bladder or vesic*) adj3 (cancer* or carcin* or malign* or tumor* or tumour* or neoplas* or papilloma*)).tw,kw. (142739)

3 exp Carcinoma, Transitional Cell/ or exp transitional cell carcinoma/ (48353)

4 ((transitional cell or urotheli*) adj3 (cancer* or carcin* or malig* or tumor* or tumour* or neoplas* or papilloma*)).tw,kw. (60950)

5 or/1-4 (180548)

6 exp Drug Therapy/ or exp chemotherapy/ or exp Hyperthermia, Induced/ (4464481)

7 (Chemohyperthermia* or Hyperthermic Intravesical Chemotherapy or Heat Targeted Drug Delivery or Chemo-hyperthermia or Hyperthermic Intravesical Instillation or HIVEC* or thermal-chemotherapy or induced hyperthermia).tw,kw. (4042)

8 6 or 7 (4467413)

9 exp BCG vaccine/tu or exp Bacillus Calmette Guerin Vaccine/tu (3987)

10 (BCG or BCG Vaccine or Bacillus Calmette Guerin Vaccine or Bacillus Calmette Guerin).tw,kw. (62661)

11 9 or 10 (63774)

12 5 and 8 and 11 (3754)

13 (exp animals/ or exp animal/ or exp nonhuman/ or exp animal experiment/ or animal model/ or animal tissue/ or non human/ or (rat or rats or mice or mouse or swine or porcine or murine or sheep or lambs or pigs or piglets or rabbit or rabbits or cat or cats or dog or dogs or cattle or bovine or monkey or monkeys or trout or marmoset$1).ti.) not (humans/ or human/ or human experiment/ or (human* or men or women or patients or subjects).tw.) (11261934)

14 (child/ or Pediatrics/ or Adolescent/ or Infant/ or adolescence/ or newborn/ or (baby or babies or child or children or pediatric* or paediatric* or peadiatric* or infant* or infancy or neonat* or newborn* or new born* or adolescen* or preschool or pre-school or toddler*).tw.) not (adult/ or aged/ or (aged or adult* or elder* or senior* or men or women).tw.) (4729928)

15 note/ or editorial/ or letter/ or Comment/ or news/ or (note or editorial or letter or Comment or news).pt. (4731791)

16 or/13-15 (19975829)

17 12 not 16 (3462)

18 limit 17 to english [Limit not valid in DARE,CLCMR,CLEED; records were retained] (2956)

19 remove duplicates from 18 (2361)

2. Risk of bias assessment for randomised controlled trials


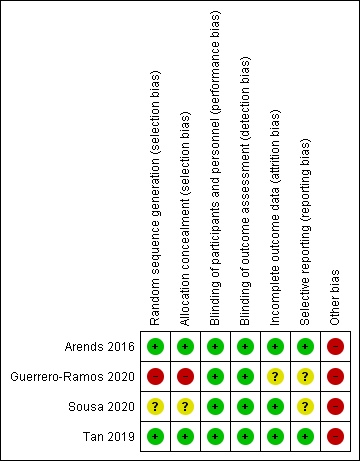


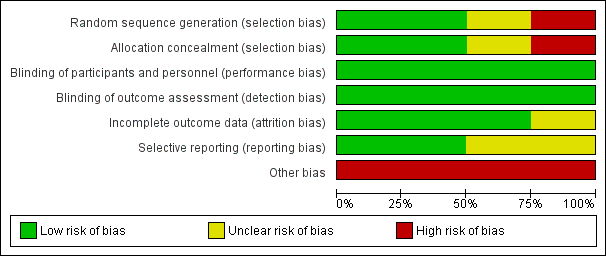


3. Risk of bias assessment for non-randomised study


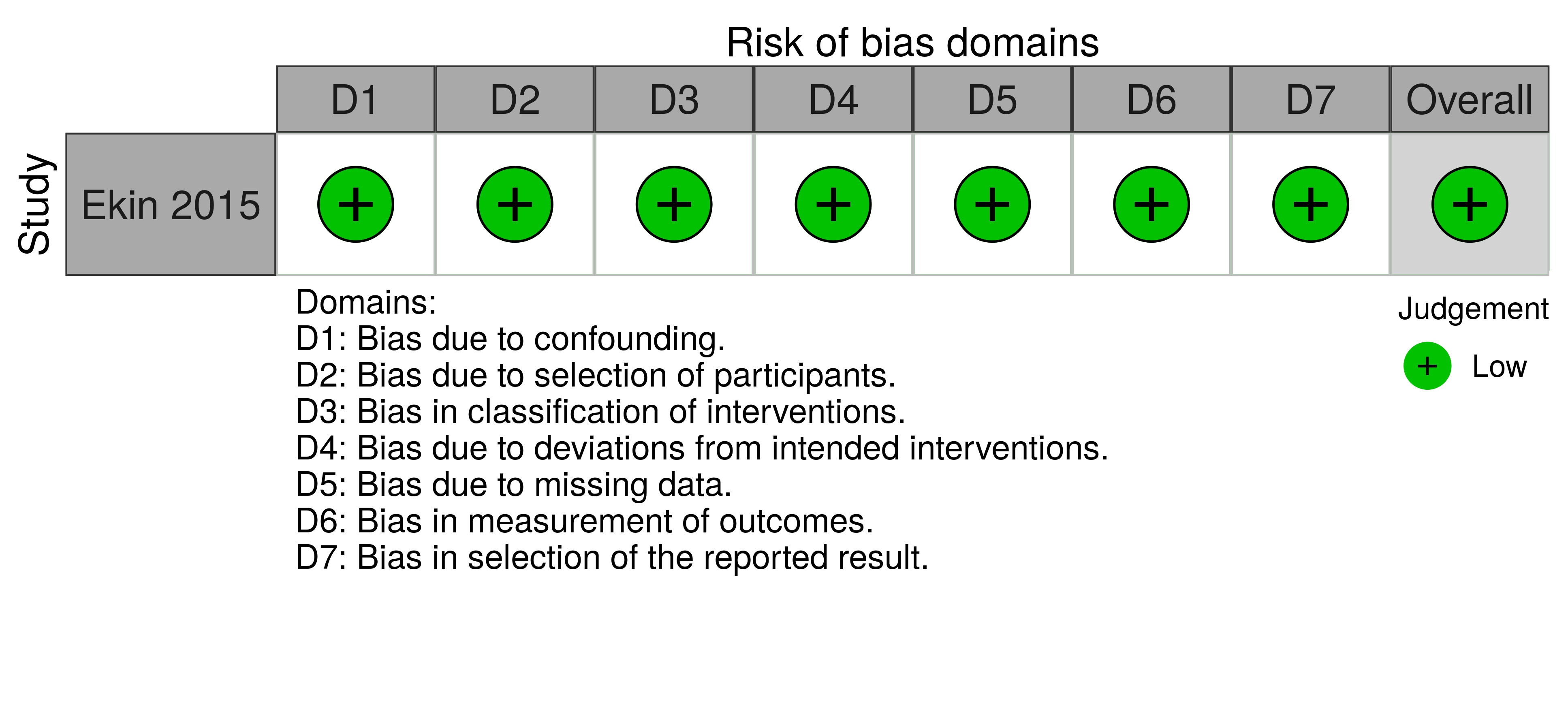


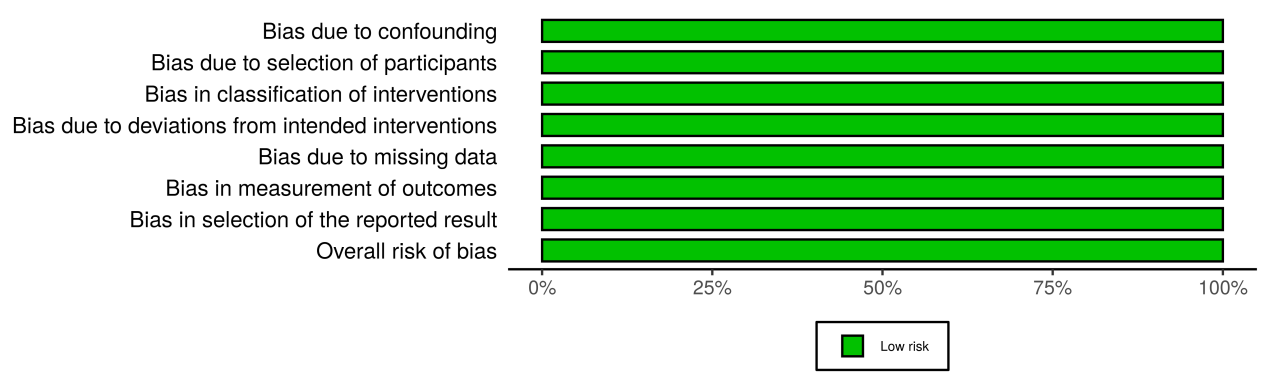


4. Recurrence-free survival stratified by the type of chemohyperthermia device


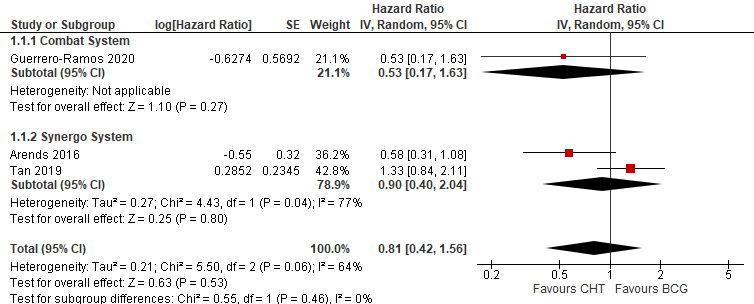


5. Progression-free survival stratified by the type of chemohyperthermia device


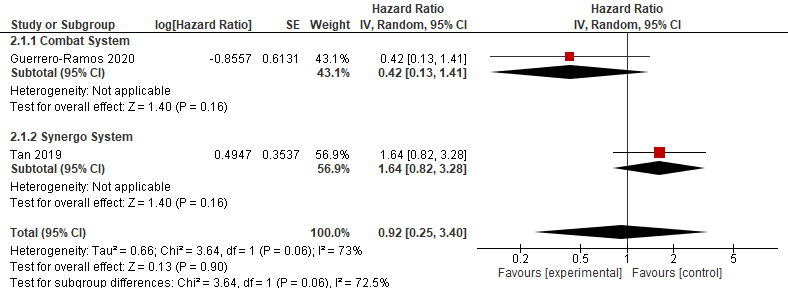


6. Recurrence rate in patients without BCG failure


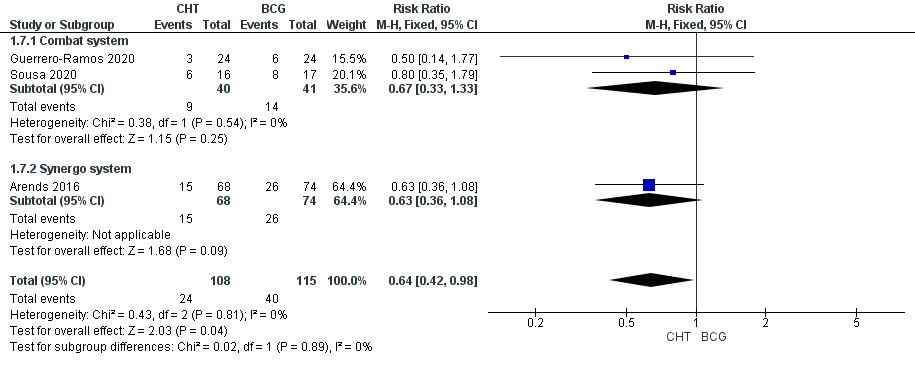


7. Progression rate in patients without BCG failure


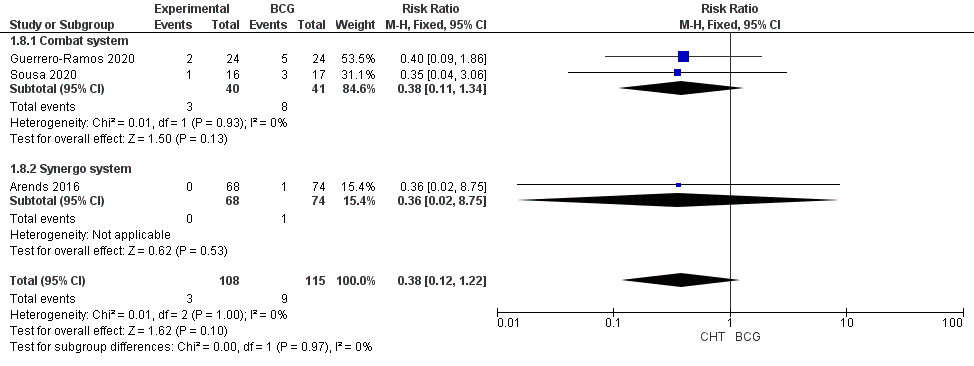


8. Recurrence-free survival in patients without BCG failure


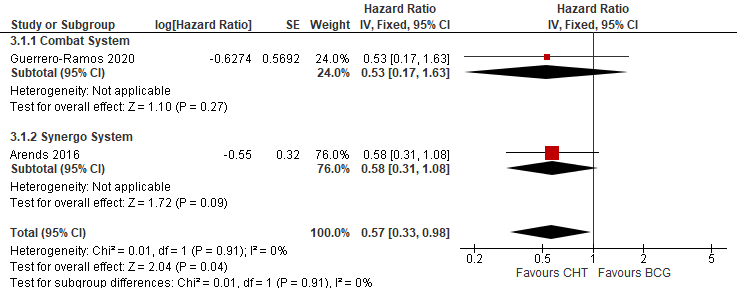


9. Recurrence-free survival in patients without CIS disease


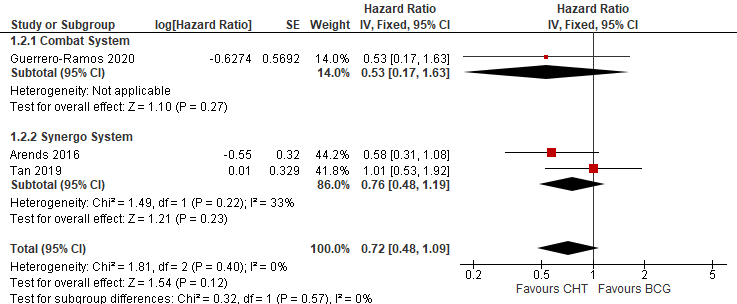


6. Grade summary of the included randomised controlled trials

| **Certainty assessment** | | | | | | | **№ of patients** | | **Effect** | | **Certainty** | **Importance** |
| --- | --- | --- | --- | --- | --- | --- | --- | --- | --- | --- | --- | --- |
| **№ of studies** | **Study design** | **Risk of bias** | **Inconsistency** | **Indirectness** | **Imprecision** | **Other considerations** | **Intravesical chemohyperthermia Instillation** | **Bacillus Calmette-Guerin instillation** | **Relative (95% CI)** | **Absolute (95% CI)** |  |  |
| **Recurrence at 24- 36 months (follow up: range 24 months to 36 months; assessed with: Risk Ratio)** | | | | | | | | | | | | |
| 4 | randomised trials | not serious | not serious | not serious | serious ^a^ | none | 46/156 (29.5%) | 64/171 (37.4%) | **RR 0.80** (0.59 to 1.08) | **75 fewer per 1,000** (from 153 fewer to 30 more) | ⨁⨁⨁◯ MODERATE | CRITICAL |
| **Progression at 24-36 months (follow up: range 24 months to 36 months; assessed with: Risk Ratio)** | | | | | | | | | | | | |
| 4 | randomised trials | not serious | not serious | not serious | very serious ^b^ | none | 7/156 (4.5%) | 13/171 (7.6%) | **RR 0.60** (0.26 to 1.41) | **30 fewer per 1,000** (from 56 fewer to 31 more) | ⨁⨁◯◯ LOW | CRITICAL |
| **Recurrence Free Survival (follow up: range 24 months to 36 months; assessed with: Hazard Ratio)** | | | | | | | | | | | | |
| 3 | randomised trials | not serious | very serious ^c^ | not serious | very serious ^b^ | none | 132 participants | 152 participants | **HR 0.81** (0.72 to 1.56) [Recurrence Free Survival] | **57 fewer per 1,000** (from 85 fewer to 142 more) | ⨁◯◯◯ VERY LOW | CRITICAL |
|  |  |  |  |  |  |  | - | 36.0% |  |  |  |  |
| **Progression Free Survival (follow up: range 24 months to 36 months; assessed with: Hazard Ratio)** | | | | | | | | | | | | |
| 2 | randomised trials | not serious | very serious ^c^ | not serious | very serious ^b^ | none | 132 participants | 152 participants | **HR 0.92** (0.25 to 3.40) [Progression Free Survival] | **6 fewer per 1,000** (from 56 fewer to 160 more) | ⨁◯◯◯ VERY LOW | CRITICAL |
|  |  |  |  |  |  |  | - | 7.6% |  |  |  |  |
| **Adverse Events Grade 1-2 (follow up: range 24 months to 36 months; assessed with: Risk Ratios)** | | | | | | | | | | | | |
| 4 | randomised trials | not serious | not serious | not serious | serious ^a^ | none | 106/177 (59.9%) | 104/191 (54.5%) | **RR 1.11** (0.93 to 1.32) | **60 more per 1,000** (from 38 fewer to 174 more) | ⨁⨁⨁◯ MODERATE | IMPORTANT |
| **Adverse Events Grade 3+ (follow up: range 24 months to 36 months; assessed with: Risk Ratio)** | | | | | | | | | | | | |
| 4 | randomised trials | not serious | not serious | not serious | very serious ^b^ | none | 41/177 (23.2%) | 43/191 (22.5%) | **RR 1.02** (0.71 to 1.47) | **5 more per 1,000** (from 65 fewer to 106 more) | ⨁⨁◯◯ LOW | IMPORTANT |

**CI:** Confidence interval; **RR:** Risk ratio; **HR:** Hazard Ratio

#### Explanations

a. Optimal information size criterion not reached

b. Wide 95% Confidence Interval includes appreciable benefit to appreciable harm

c. High heterogeneity observed among studies

7. Grade summary of the included non-randomised study

| **Certainty assessment** | | | | | | | **№ of patients** | | **Effect** | | **Certainty** | **Importance** |
| --- | --- | --- | --- | --- | --- | --- | --- | --- | --- | --- | --- | --- |
| **№ of studies** | **Study design** | **Risk of bias** | **Inconsistency** | **Indirectness** | **Imprecision** | **Other considerations** | **[intervention]** | **[comparison]** | **Relative (95% CI)** | **Absolute (95% CI)** |  |  |
| **Recurrence Rate (follow up: median 33 months; assessed with: Risk Ratio)** | | | | | | | | | | | | |
| 1 | observational studies | not serious | not serious | not serious | serious ^a^ | none | 14/40 (35.0%) | 8/142 (5.6%) | **RR 6.21** (2.80 to 13.75) | **294 more per 1,000** (from 101 more to 718 more) | ⨁⨁⨁◯ MODERATE | CRITICAL |
| **Progression Rate (follow up: median 33 months; assessed with: Risk Ratio)** | | | | | | | | | | | | |
| 1 | observational studies | not serious | not serious | not serious | serious ^a^ | none | 6/40 (15.0%) | 3/142 (2.1%) | **RR 6.7619** (1.7662 to 25.8884) | **122 more per 1,000** (from 16 more to 526 more) | ⨁⨁⨁◯ MODERATE | CRITICAL |
| **Recurrence Free-Survival (follow up: median 33 months; assessed with: Hazard Ratio)** | | | | | | | | | | | | |
| 1 | non-randomised studies | not serious | not serious | not serious | serious ^a^ | none | 39 participants | 39 participants | **HR 4.18** (1.37 to 12.71) [Recurrence Free-Survival] | **412 more per 1,000** (from 65 more to 741 more) | ⨁⨁⨁◯ MODERATE | CRITICAL |
|  |  |  |  |  |  |  | - | 20.5% |  | **412 more per 1,000** (from 65 more to 741 more) |  |  |
| **Progression Free Survival (follow up: median 33 months; assessed with: Hazard Ratio)** | | | | | | | | | | | | |
| 1 | non-randomised studies | not serious | not serious | not serious | very serious ^b^ | none | 39 participants | 39 participants | **RR 1.72** (0.28 to 10.36) [Progression Free Survival] | **55 more per 1,000** (from 55 fewer to 720 more) | ⨁⨁◯◯ LOW | CRITICAL |
|  |  |  |  |  |  |  | - | 7.7% |  | **55 more per 1,000** (from 55 fewer to 720 more) |  |  |

**CI:** Confidence interval; **RR:** Risk ratio; **HR:** Hazard Ratio

#### Explanations a. Wide 95% CI b. Wide 95% CI including appreciable benefits and harm
